# Power Flow in Multimode Graded-Index Microstructured Polymer Optical Fibers

**DOI:** 10.3390/polym15061474

**Published:** 2023-03-16

**Authors:** Svetislav Savović, Ana Simović, Branko Drljača, Milan S. Kovačević, Ljubica Kuzmanović, Alexandar Djordjevich, Konstantinos Aidinis, Rui Min

**Affiliations:** 1 Faculty of Science, University of Kragujevac, R. Domanovića 12, 34000 Kragujevac, Serbia; 2 Department of Mechanical Engineering, City University of Hong Kong, 83 Tat Chee Avenue, Hong Kong, China; 3 Faculty of Sciences, University of Priština in Kosovska Mitrovica, Lole Ribara 29, 38220 Kosovska Mitrovica, Serbia; 4 Department of Electrical Engineering, Ajman University, Ajman P.O. Box 346, United Arab Emirates; 5 Center of Medical and Bio-Allied Health Sciences Research, Ajman University, Ajman P.O. Box 346, United Arab Emirates; 6 Center for Cognition and Neuroergonomics, State Key Laboratory of Cognitive Neuroscience and Learning, Beijing Normal University at Zhuhai, Zhuhai 519087, China

**Keywords:** polymer optical fiber, graded-index optical fiber, microstructured optical fiber, power flow equation

## Abstract

We investigate mode coupling in a multimode graded-index microstructured polymer optical fiber (GI mPOF) with a solid core by solving the time-independent power flow equation (TI PFE). Using launch beams with various radial offsets, it is possible to calculate for such an optical fiber the transients of the modal power distribution, the length *L_c_* at which an equilibrium mode distribution (EMD) is reached, and the length *z_s_* for establishing a steady-state distribution (SSD). In contrast to the conventional GI POF, the GI mPOF explored in this study achieves the EMD at a shorter length *L_c_*. The earlier shift to the phase of slower bandwidth decrease would result from the shorter *L_c_*. These results are helpful for the implementation of multimode GI mPOFs as a part of communications and optical fiber sensory systems.

## 1. Introduction

High-speed, short-range signal transmission over POF has attracted much research interest in recent years [1,2]. POF has the benefit of an easy connection and a large core, which could be an economic solution for the in-home network. Different kinds of materials are implemented for POF fabrication, such as polymethyl methacrylate (PMMA) [3,4], polydimethylsiloxane (PDMS) [5,6], polycarbonate (PC) [7,8], polystyrene (PS) [9,10], perfluorinated polymer (commercially known as CYTOP^®^ (AGC, Inc., Tokyo, Japan) [11,12], cycloolefin polymer (commercially known as ZEONEX^®^ *ZEON (Corporation, Tokyo, Japan)) [13,14], and cycloolefin copolymer (commercially known as TOPAS^®^) (TOPAS Advanced Polymers, Farmington Hills, MI, USA) [15,16]. The flexibility of POF material allows for the production of POFs with varying specifications or materials to meet the needs of various applications. Until now, PMMA has been the most commonly used material for the production of POF [17]. POF can normally be classified as singlemode [18] or multimode [19] based on the number of propagation modes, the step-index (SI) [20], or the GI [21] based on the refractive index (RI) distribution. The GI multimode POF is a type of POF where its RI distribution decreases continuously from the core axis to the cladding. This RI distribution can minimize intermodal dispersion, improve the POF’s bandwidth, and increase the transmission distance. However, GI POF needs sophisticated doping processes for its fabrication.

**Figure 1:**
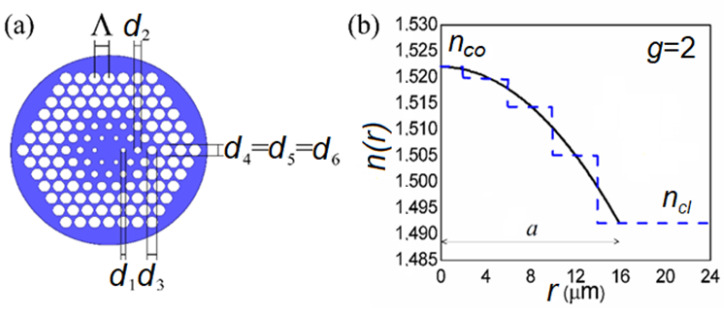
(**a**) The multimode GI MOF’s cross-section. Pitch Λ is used to position the air holes in a triangular lattice. The four inner air-hole rings in the core have the following air-hole diameters: *d*_1_, *d*_2_, *d*_3_, and *d*_4_. The diameter of the air holes in the cladding in rings 5 and 6 is the same as that in air-hole ring 4 (*d*_4_ *= d*_5_ = *d*_6_). (**b**) The RI performance of the referent multimode GI MOF (blue dashed line). The RI in the core has a parabolic distribution (1) with *g* = 2 (black solid line).

Microstructured optical fiber (MOF), which is referred to as photonic crystal fiber, was successfully proposed in the 1990s [22]. The microstructure of MOFs significantly improves the optical fiber’s flexibility. By adjusting the microstructure, various excellent MOF features have been explored, such as birefringence [23], light dispersion [24], supercontinuum light [25], and wavelength conversion [26]. Argyros developed the first PMMA mPOF in 2001 [27], and then mPOF attracted research interests for its different applications [28,29]. The core and/or cladding layer of a typical mPOF design, as depicted in Figure 1, can be changed by altering the arrangement and/or size (*d*) of air holes within a concentric ring-like region. Figure 1 depicts an mPOF that mimics a GI optical fiber by having a core with varying sizes of air-holes. Greater flexibility in modifying the air-hole diameters and pitches, as opposed to the necessity for sophisticated doping processes with typical GI POF, is the advantage of GI mPOF over conventional GI POF. Additionally, GI mPOF has been found to have a higher bandwidth and a lower loss than conventional GI POF [30].

The performance of GI mPOF is substantially impacted by mode coupling. Light scattering, which happens when random anomalies in multimode optical fibers transfer power from one mode to another, is the main cause of mode coupling. Power distribution changes as fiber length increases until an EMD is created at “coupling length” *L_c_*. The coupling length *L_c_* at which EMD is achieved marks the fiber length at which the highest-order guiding mode shifted its distribution to *m* = 0. Beyond *L_c_*, the light is equally distributed, and the coupling process is essentially complete. With the creation of the SSD, every distribution that is launched has a unique disc far-field pattern. In other words, length *z_s_* designates the fiber length at which the distribution of the output angular power is entirely independent of the launch beam. Transmission bandwidth is increased, and modal dispersion is decreased via mode coupling [30]. It is also interesting to note that until the SSD has been entirely obtained, the fundamental optical characteristics of an optical fiber, such as attenuation and bandwidth, cannot be precisely characterized due to mode coupling. So, knowledge of the fiber length at which an SSD is established is essential.

Up until recently, there were no commercial simulation tools available for studying the transmission characteristics of multimode MOFs. To overcome this problem, this research numerically solves the TI PFE to characterize light transmission in GI mPOF. We determined lengths for accomplishing the EMD and SSD for multimode GI mPOF with a solid core utilizing launch beam distributions with various radial offsets. We assumed that the core’s and cladding’s air holes are arranged in a series of triangles with a regular pitch Λ (see Figure 1). According to our best knowledge, this research is the first to investigate how mode coupling affects the power flow in mPOF with a GI refractive index distribution.

## 2. GI mPOF Design

Figure 1 depicts a GI mPOF considered in this study. The GI mPOF has six air-hole rings, which are designated as rings from 1 to 6.

A polymer is taken into consideration as the fiber material, and a triangular lattice with a pitch Λ is used to hold the air holes. The four inner air-hole rings in the core provide a parabolic RI distribution due to the selected air-hole diameter distribution. The air-hole diameter in rings 5 and 6 is the same as the air-hole diameter in ring 4 (*d*_4_ = *d*_5_ = *d*_6_). This system was simulated using the TI PFE.

## 3. Time-Independent Power Flow Equation

The refractive index profile of GI optical fibers is given as:

(1) $$n(r,\lambda )=\left\{\begin{array}{l} n_{co}(\lambda )\;{\left[1-2\Delta (\lambda ){\left(\frac{r}{a}\right)}^{g}\right]}^{1/2}\;\;\;\;\;\;\;\;(0\leq r\leq a)\\ n_{co}(\lambda )\;{\left(1-2\Delta (\lambda )\right)}^{1/2}\;\;=n_{cl}\left(\lambda \right)\;\;\;\;\;\;\;\;\;(r>a) \end{array}\right.\;$$

where *g* is the core index exponent, *a* is the core radius, *n_co_*(*λ*) is the core’s highest index (measured at the fiber axis), *n_cl_*(*λ*) is the cladding’s index, and Δ = [*n_co_*(*λ*) − *n_cl_*(*λ*)]/*n_co_*(*λ*) is the relative index difference.

The TI PFE for a GI optical fiber is [31]:

(2) $$\frac{\partial P(m,\lambda ,z)}{\partial z}=\frac{D}{m}\frac{\partial P(m,\lambda ,z)}{\partial m}+D\frac{\partial P^{2}(m,\lambda ,z)}{\partial m^{2}}\;$$

where *P*(*m*,*λ*,*z*) is the power in the *m*-th principal mode (modal group), *z* is the coordinate along the fiber axis, and *D* is a constant mode coupling coefficient. The maximum principal mode number *M*(*λ*) can be obtained as [31]:

(3) $$\;\;M(\lambda )=\sqrt{\frac{g\Delta (\lambda )}{g+2}}akn_{co}(\lambda )\;$$

where *k* = 2*π/λ*.

Using the explicit finite difference method, the discretization of Equation (2) leads to [31]:

(4) $$\begin{array}{l} P_{i,j+1}=\left(\frac{D\Delta z}{{\left(\Delta m\right)}^{2}}-\frac{D\Delta z}{2m_{i}\Delta m}\right)P_{i-1,j}+\left(1-\frac{2D\Delta z}{{\left(\Delta m\right)}^{2}}\right)P_{i,j}\\ \;\;+\left(\frac{D\Delta z}{2m_{i}\Delta m}+\frac{D\Delta z}{{\left(\Delta m\right)}^{2}}\right)P_{i+1,j} \end{array}$$

where *i* and *j* refer to the discretization step lengths Δ*m* and Δ*z* for the mode *m* and length *z*, respectively. This work reports, to the best of the authors’ knowledge, the first solution of Equation (4) of the TI PFE Equation (2) for an investigation of mode coupling along a GI mPOF in terms of mode variable (*m*).

The principal mode *m* excited at the input fiber end is [31]:

(5) $$\frac{m}{M}={\left[{\left(\frac{\Delta r}{a}\right)}^{g}+\frac{\theta^{2}}{2\Delta }\right]}^{(g+2)/2g}$$

where *θr* is the launch beam radial offset and *θ* is the launch beam angle.

## 4. Numerical Simulation Results

The light transmission in a multimode GI mPOF with a solid core (Figure 1) was investigated. For such a fiber, the effective *V* parameter is given as:

(6) $$V=\frac{2\pi }{\lambda }a_{eff}\sqrt{n_{0}^{2}-n_{fsm}^{2}}$$

where *a_eff_* = $\Lambda /\sqrt{3}$ [32], and *n_fsm_* is the effective RI of different core and cladding layers, which are obtained from Equation (6), with the effective *V* parameter [33]:

(7) $$V\left(\frac{\lambda }{\Lambda },\frac{d}{\Lambda }\right)=A_{1}+\frac{A_{2}}{1+A_{3}\exp\left(A_{4}\lambda /\Lambda \right)}$$

with the fitting parameters *A_i_* (*i* = 1 to 4) as below:

(8) $$A_{i}=a_{i0}+a_{i1}{\left(\frac{d}{\Lambda }\right)}^{b_{i1}}+a_{i2}{\left(\frac{d}{\Lambda }\right)}^{b_{i2}}+a_{i3}{\left(\frac{d}{\Lambda }\right)}^{b_{i3}}$$

where the coefficients from *a*_*i*0_ to *a*_*i*3_ and from *b*_*i*1_ to *b*_*i*3_ (*i* = from 1 to 4) are given in Table 1.

We applied our method to the GI mPOF, which has a core radius of *a* = 4Λ = 16 µm, where Λ = 4 μm, and a fiber diameter of *b* = 1 mm. Measured along the fiber axis, the core’s refractive index is *n_co_* = 1.5220 and the refractive index of the cladding is *n_cl_* = 1.4920 [29]. The maximum principal mode number for the GI mPOF under study is *M* = 580 at λ = 633 nm, for *g* = 2.0, and $∆=(n_{co}-n_{cl})/n_{co}$ = 0.019711. The coupling coefficient is *D* = 1482 1/m [29] (the typical value of *D* for GI mPOFs and conventional GI POFs). Because the strength of mode coupling in both conventional GI POFs and GI mPOFs is correlated with the polymer core material, it is significant to highlight that while modeling the GI mPOF, the typical values of *D* that describe a conventional GI POF can be utilized. In modeling a silica MOF, the same assumption was made [31]. For Λ = 4 μm and the air-hole diameters of the four air-hole rings in the core, *d*_1_ = 0.6 μm, *d*_2_ = 0.7 μm, *d*_3_ = 1.3 μm, and *d*_4_ = 3.1 μm, the refractive indexes *n*_1_ = 1.5201, *n*_2_ = 1.5145, *n*_3_ = 1.5050, and *n*_4_ = 1.4920, respectively, are calculated utilizing Equations (6) and (7). Thus, a parabolic RI distribution in the core with *g* = 2.0 is achieved (Figure 1). The diameter of the cladding’s air-holes in rings 5 and 6 is *d*_4_ = *d*_5_ = *d*_6_ = 3.1 μm, which corresponds to the cladding refractive index *n*_4_ = *n*_5_ = *n*_6_ = *n_cl_* = 1.4920.

**Figure 2:**
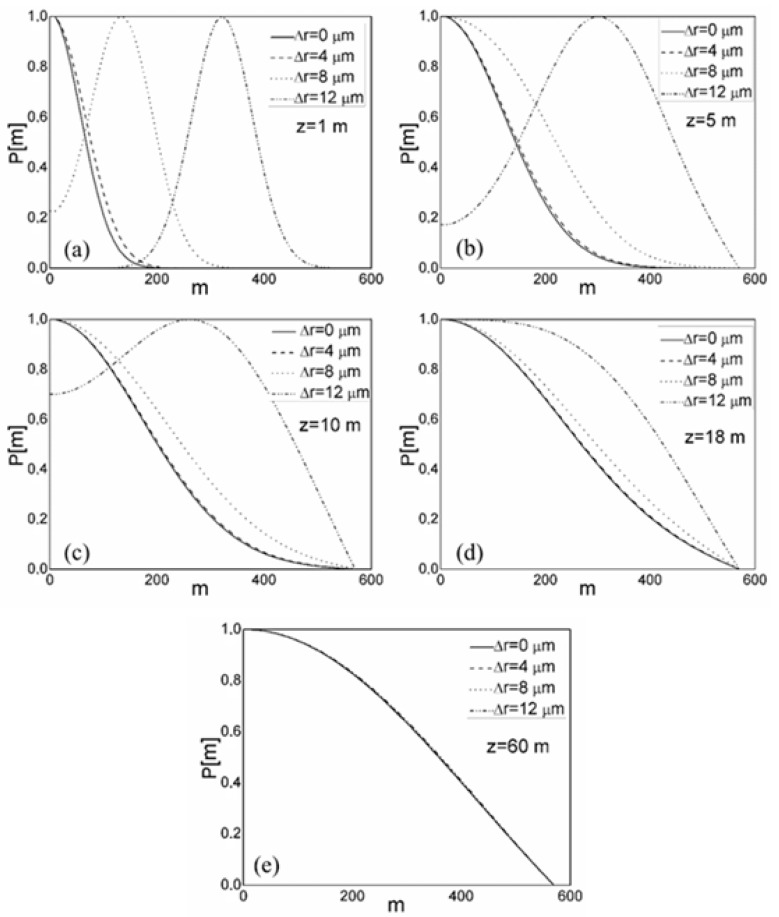
Normalized output modal power distribution *P*(*m*,*λ*,*z*) over a range of radial offsets Δ*r* obtained by numerically solving the TI PFE (2) at different fiber lengths: (**a**) *z* = 1 m, (**b**) *z* = 5 m, (**c**) *z* = 10 m, (**d**) *z* = 18 m, and (**e**) *z* = 60 m.

Figure 2 depicts the evolution of the fiber length-dependent normalized output modal power distribution *P*(*m*,λ,*z*). The numerical calculations assume a Gaussian beam *P*(*θ*,*z*) launched with $\langle \theta \rangle$ = 0° (Equation (5)). The results are shown for four different radial offsets, ∆r = 0, 4, 8, and 12 µm. Low-order modes’ coupling is stronger in short fibers (their modal distributions have already shifted towards *m* = 0). Due to the transfer of optical power during transmission from lower- to higher-order modes, higher-order modes can only be coupled with longer fiber lengths. The midpoints of all modes’ power distributions have been moved to zero (*m* = 0) at the coupling length of *L_c_* = 18 m, resulting in the EMD, Figure 2d. SSD is established at *z* ≡ *z_s_* = 60 m. It should be noted that a coupling length of *L_c_* = 31 m is reported for a conventional GI POF, which had the same coupling coefficient *D* = 1482 1/m and was investigated in our previous work [31]. The smaller core radius and consequently fewer propagating modes in the GI mPOF than in a conventional GI POF (the maximum principal mode number for a conventional GI POF was *M* = 656) result in a shorter coupling length in the former. To put it another way, fewer propagating modes need to couple together for a shorter length. Silica MOFs have significantly less mode coupling than the GI mPOF that was the subject of this study and, therefore, much longer lengths *L_c_* from ≃ 1.45 to 1.65 km at which an EMD is achieved and length *z_s_* from ≃ 3.30 to 3.80 km at which an SSD is established [33]. One should note that in modeling optical fibers with a GI distribution, it is commonly assumed that *D* is constant, i.e., it is independent of *m* [31]. The same assumption of mode-independent *D* is also used in modeling step-index microstructured polymer optical fibers [33,34].

It is significant to note that the length dependence of the GI MOF bandwidth is determined by mode coupling behavior. A length below the coupling length *L_c_* has an inverse linear effect on the bandwidth. However, it has a *z*^−1/2^ dependence beyond this equilibrium length *L_c_.* The faster shift to the phase of slower bandwidth decrease would therefore result from the shorter *L_c_* [29,34]. A quicker bandwidth enhancement in the examined mPOF is expected compared to conventional GI POFs, demonstrating that GI mPOFs are a superior option for short-range telecommunication lines since the lengths needed to establish an EMD and SSD in GI mPOFs are less than in conventional GI POFs [31].

It is obvious that forcing a mode coupling process results in faster bandwidth improvement. In practice, mode coupling can be increased using an appropriate scrambler, which can be installed near the input fiber end. This would lead to a significant decrease in coupling length *L_c_* and, therefore, a faster bandwidth improvement (a slower bandwidth decrease).

The findings obtained in this research can be used in a variety of communication and sensory systems that utilize multimode GI mPOFs. The ability to identify the modal distribution of the GI mPOF used as a component of the optical fiber sensory system at a specific length is also crucial. The theoretical approach of modal diffusion in MOFs utilized in this study can be used to calculate the bandwidth of a fiber; however, one must solve the time-dependent power flow equation [35] rather than the TI PFE (2) that was used in this work.

## 5. Conclusions

The TI PFE used to investigate the state of mode coupling along a GI mPOF has a numerical solution reported in this work. As a result of a strong mode coupling process, which is common for polymer optical fibers, the results demonstrate that the coupling lengths for obtaining the EMD and the lengths for establishing SSD are short in this fiber. Due to the significant intrinsic perturbation effects in the GI mPOF, such strong mode coupling is explained. In comparison to a conventional GI POF, the EMD in the GI mPOF examined in this study is attained at an even shorter length, *L_c_*, i.e., the coupling length *L_c_* is reduced by a factor of 1.7 (*L_c_* = 18 m in GI mPOFs as compared to *L_c_* = 31 m in conventional GI POFs). This is because the GI mPOF has a smaller core radius and, thus, a smaller number of propagating modes. Namely, a smaller number of propagating modes necessitates a shorter length to complete the mode coupling process. As a result, the shorter *L_c_* leads to a faster transition to the slower bandwidth regime. For their employment in data transmission, power supply, sensing, and other systems that could be impacted by variations in power quality, the fiber characterization given in this work is essential.

## Figures and Tables

**Table 1:** The fitting coefficients in Equation (8).

	*i* = 1	*i* = 2	*i* = 3	*i* = 4
*a*_*i*0_	0.54808	0.71041	0.16904	−1.52736
*a*_*i*1_	5.00401	9.73491	1.85765	1.06745
*a*_*i*2_	−10.43248	47.41496	18.96849	1.93229
*a*_*i*3_	8.22992	−437.50962	−42.4318	3.89
*b*_*i*1_	5	1.8	1.7	−0.84
*b*_*i*2_	7	7.32	10	1.02
*b*_*i*3_	9	22.8	14	13.4

## Data Availability

The data presented in this study are available on request from the corresponding author.
