# Correction: Savović et al. Power Flow in Multimode Graded-Index Microstructured Polymer Optical Fibers. *Polymers* 2023, *15*, 1474

**DOI:** 10.3390/polym15112553

**Published:** 2023-06-01

**Authors:** Svetislav Savović, Ana Simović, Branko Drljača, Milan S. Kovačević, Ljubica Kuzmanović, Alexandar Djordjevich, Konstantinos Aidinis, Rui Min

**Affiliations:** 1 Faculty of Science, University of Kragujevac, R. Domanovića 12, 34000 Kragujevac, Serbia; savovic@kg.ac.rs (S.S.); asimovic@kg.ac.rs (A.S.); kovac@kg.ac.rs (M.S.K.); ljubica.kuzmanovic@pmf.kg.ac.rs (L.K.); 2 Department of Mechanical Engineering, City University of Hong Kong, 83 Tat Chee Avenue, Hong Kong, China; mealex@cityu.edu.hk; 3 Faculty of Sciences, University of Priština in Kosovska Mitrovica, Lole Ribara 29, 38220 Kosovska Mitrovica, Serbia; branko.drljaca@pr.ac.rs; 4 Department of Electrical Engineering, Ajman University, Ajman P.O. Box 346, United Arab Emirates; k.aidinis@ajman.ac.ae; 5 Center of Medical and Bio-Allied Health Sciences Research, Ajman University, Ajman P.O. Box 346, United Arab Emirates; 6 Center for Cognition and Neuroergonomics, State Key Laboratory of Cognitive Neuroscience and Learning, Beijing Normal University at Zhuhai, Zhuhai 519087, China

The authors wish to make three changes to their published paper [1].

There is a mistake in Section 3, on page 4. The value of the maximum principal mode number *M* was wrong, and the value of the maximum mode number *N* was missing. The corrected sentence is shown below:

The maximum mode number for the GI mPOF under study is *N* = 580 and the maximum principal mode number is *M* = *N*^1/2^ = 24 at λ = 633 nm, for *g* = 2.0, and $∆=\left(n_{co}-n_{cl}\right)/n_{co}$ = 0.019711.

There is a mistake in Figure 2. The *X*-axis label should be *m*/*M*. The authors added “(the maximum principal mode number is *M* = 24; the maximum mode number is *N* = 580)” in the legend for Figure 2. The corrected Figure 2 appears below. 

The authors state that the scientific conclusions are unaffected. This correction was approved by the Academic Editor. The original publication has also been updated.

## Figures and Tables

**Figure 2:**
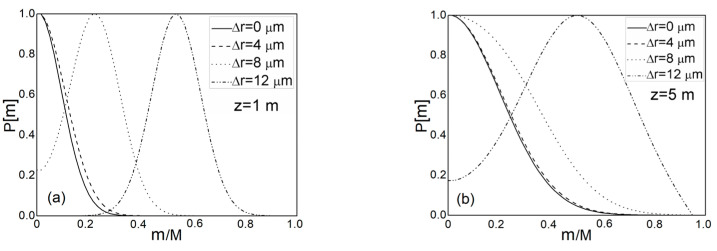

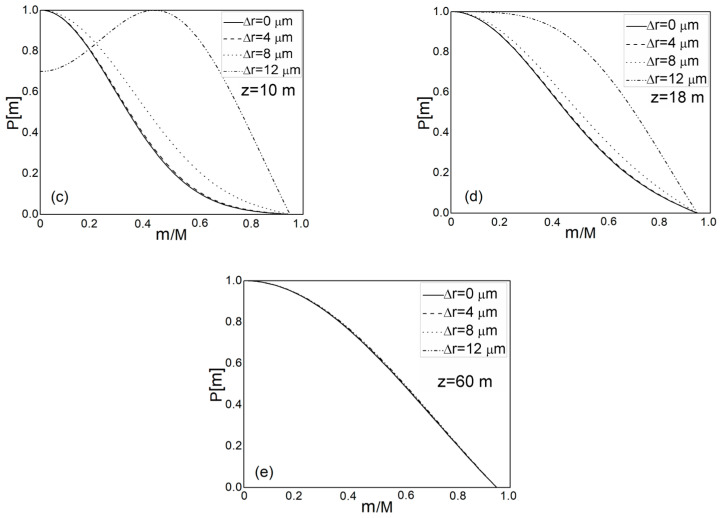
Normalized output modal power distribution *P*(*m*,*λ*,*z*) over a range of radial offsets ∆r obtained by numerically solving the TI PFE (2) at different fiber lengths: (**a**) *z* = 1 m, (**b**) *z* = 5 m, (**c**) *z* = 10 m, (**d**) *z* = 18 m, and (**e**) *z* = 60 m (the maximum principal mode number is *M* = 24; the maximum mode number is *N* = 580).

## References

[1] Savović S., Simović A., Drljača B., Kovačević M.S., Kuzmanović L., Djordjevich A., Aidinis K., Min R. (2023). Power flow in multimode graded-index microstructured polymer optical fibers. Polymers.

